# Accessing digital harm reduction services —exploring the impact of the “Here4UScotland” application

**DOI:** 10.1186/s12954-026-01418-w

**Published:** 2026-02-16

**Authors:** Graeme Strachan, Hadi Daneshvar, Catriona Matheson

**Affiliations:** 1 School of Law, University of Sheffield, Sheffield, UK; 2 School of Health and Social Care, Edinburgh Napier University, Edinburgh, UK; 3 Centre for Healthcare and Community Research, University of Stirling, Stirling, UK

**Keywords:** Digital harm reduction, Drug-related-deaths, Qualitative research, Service users, Service providers, Visual cues

## Abstract

**Background:**

The unique challenges faced by vulnerable drug users highlight the urgent need for accessible, immediate digital interventions which people can access anywhere and at any time. This study explores the impact of the Here4UScotland virtual supervised consumption app, examining relationships between service users and providers, their separate relationships with harm reduction digital solutions and the app’s effects on personal and collaborative service engagement.

**Methods:**

The “Here4UScotland” app was piloted in Aberdeen, Scotland between May 2022 to Aug 2023, which incorporates its gestational inception to front-end live engagement. This qualitative study employed two focus groups (*n* = 8)). These were conducted independent of semi-structured interviews (*n* = 21) which individually investigated the various experiences of service users, supporters, and stakeholders. In total 26 people provided data which was thematically analysed using NVivo 12 to look for associated and relevant codes and themes using the Technology, People, Organisational, and Macro-environmental (TPOM) framework.

**Results:**

Main technology themes were video calling, location and privacy and usability/connection. Under ‘people’ positive relationships, finding identify and enhanced digital safety were described. Organisational themes covered ways to cultivating trust, supporter’s responsibility, and associated training for services and supporters. Key concerns emerged regarding the absence of crucial visual cues for staff and the potential for police involvement.

**Conclusions:**

Digital interventions like Here4UScotland offer significant benefits in enhancing harm reduction engagement and access, fostering new connections and community among vulnerable populations. Digital access beyond virtual consumption was considered. For successful integration of this technology, it appears crucial to balance technological advantages with ensuring privacy, providing adequate training for staff, and integrating these solutions with existing services, rather than replacing essential human interaction.

## Introduction

Worldwide drug-related deaths have become a public health crisis, intensified by synthetics like fentanyl and nitazenes in the street supply [1, 2]. North America reported 81,083 opioid-related deaths in 2023, disproportionately affecting marginalised communities [3–5]. Canada saw 7162 drug-related deaths in 2023, with British Columbia reporting 2511 [6–8]. The European Monitoring Agency noted 74% of 2021 drug-related deaths were due to opioids [2].

In 2024 Scotland registered 1017 drug fatalities which was an encouraging decrease from the 1172 drug deaths registered in 2023; however, the country maintains its status as Europe’s drug death capital [9]. Opioids feature in 80% of deaths, but polydrug use is common (81% of all deaths), with benzodiazepines in 58%, cocaine (41%) and gabapentinoids in 38% [10]. The most socio-economically deprived areas, experience 15 times more fatalities [10]. Current advice includes not using alone, engaging with needle exchange and carrying naloxone [11, 12]. However, physical services pose logistical challenges for those unable or unwilling to travel to city centre locations or engage in one-to-one communication without anonymity [13]. Vulnerable groups often feel intimidated, apprehensive, or excluded when contacting health services due to negative social perception and resulting stigma [14–16]. These global trends and a public health emergency underscore Scotland’s need for unique, effective, and immediately accessible digital harm reduction interventions to address critical public health challenges. The study aimed not only to explore the perceived impact of the Here4UScotland service but also to understand participants’ wider experiences of the app and its functionality and usability.

## Digital harm reduction

Embracing digital evolution offers a potential solution through more transient mediums and services that can assist social anxieties [17]. Digital access can navigate privacy concerns, providing safe, secure, and private connections to supervised consumption and community support for isolated individuals [18]. Remotely supervised consumption is internationally successful via accessible telephone hotlines and digital applications connecting to experienced, peer-led harm reduction support and signposting [19–21]. Providers like Safespot (formerly Never Use Alone) have operated in the USA since 2020 [19], and Canada’s National Overdose Response Service (NORS) has offered a telephone hotline since April 2020 [20]. Daneshvar and colleagues reviewed digital applications, which uses a timer for overdose alerts [22]. These apps monitor users and alert supporters or emergency services when needed. Typically, users activate the app before drug use; if they fail to respond after a set time, the app connects them to help. Some also offer live communication with trained supporters who can coordinate a rescue or contact emergency services (e.g., Brave). The Brave app, based in Canada, operated as a 24/7 remote supervised consumption service providing anonymous peer support and emergency response but was discontinued in January 2025. Less formal digital services, such as peer spotting via friends/family or user groups, also proved successful in the provinces of Ontario and Nova Scotia. These informal and formal spotting networks were investigated by the University of Toronto in a qualitative study which found that, although useful for remote groups or people who could not attend, it was not a replacement for physical services [23]. Investigations into further digital service evolution include access to oxygen measurements, vital signs, and respiratory rate for more substantial health data [22].

Digital solutions provide immediate, fluid access to previously static traditional services, fostering new relationships and trustthrough phone or video calls [24–26]. This shift from physical to multi-dimensional digital mediums navigates logistical issues, affording access to anyone with a digital device and internet. It promotes an interconnected, symbiotic relationship between harm reduction services and their users [26].

### The current study

The Here4UScotland application, piloted in Aberdeen (Scotland), introduced local drug users to accessible remote supervision [25–27]. This formative service based on the Brave overdose response app encouraged users to contact a supporter via smartphone for supervisory contact during drug use [28]. The app allowed people to talk to trained supporters by audio connection through the app while they were preparing, using and consuming drugs. These crucial periods before, during and after drug use could be remotely supported to reduce negative health or social outcomes. In addition to supervision, an emergency plan was arranged to protect individuals from potential overdose should a person become unresponsive. However, this plan deviated from other north American models (the Brave app) in that it insisted that an ambulance must be called. This was prohibitive for people as in Scotland this automatically triggers police attendance at the scene.

This study explores how the introduction of the Here4UScotland remotely supervised consumption service, and the smartphones provided to enable access to it, shaped the everyday experiences of people who use drugs, supporters and local stakeholders in Aberdeen. Using the TPOM (Technology, People, Organisation, Macro-environment) framework, we sought to understand (i) how participants experienced and made sense of the technology, (ii) how the service influenced relationships, engagement with harm reduction support and feelings of safety, and (iii) what organisational and wider contextual factors enabled or constrained implementation. Rather than evaluating clinical effectiveness, our aim was to build on previous research and generate an in-depth qualitative account of the perceived benefits, limitations and unintended consequences of this formative digital harm reduction intervention [25].

## Methodology

The Here4UScotland programme was available in Aberdeen from Jan-Aug 2024 There were 25 smartphones provided to some of those most vulnerable and at risk through the Digital Lifelines Scotland programme and partnership with Alcohol & Drug Action (ADA) in Aberdeen. These smartphones were provided with internet connectivity and prepaid credit to ensure participants could engage with the Here4UScotland app. A mixed design approach was employed with twenty-one interviews (*n* = 21) being completed but some being repeated (at different times) with the same participants to investigate and compare temporal experiences related to app engagement. In addition, two focus groups were conducted (*n* = 2). In total twenty-three (*n* = 23) recordings were recovered for analysis.

## Theoretical framework

**Fig. 1:**
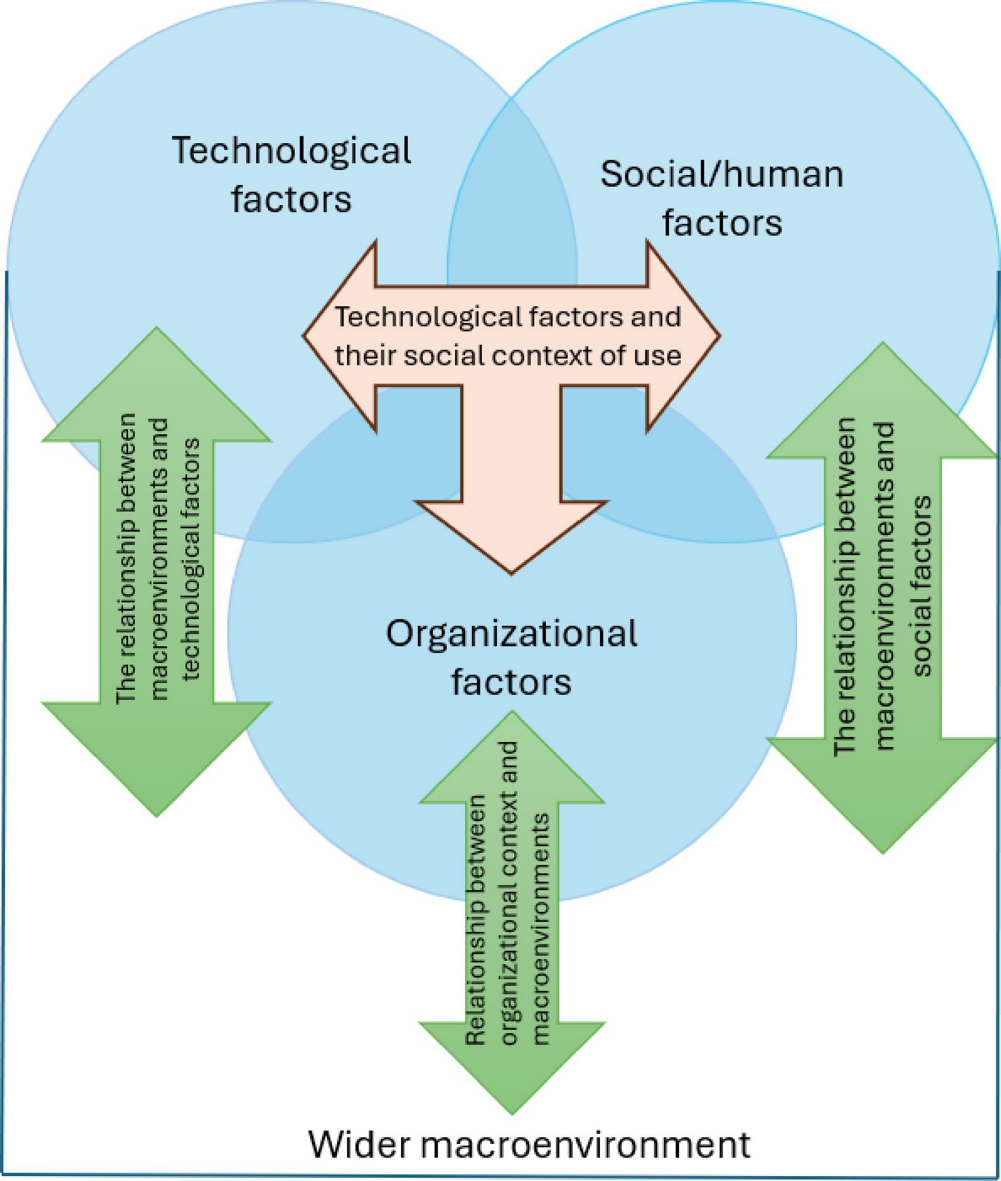
Diagram of the TPOM evaluation framework

The study applied the very practical Technology, People, Organisational, and Macro-environmental (TPOM) framework [29], developed to support the implementation and adoption of technology into health services by addressing four key domains and related sub-themes. Its focus on technological, social, organisational, and macro-environmental factors supports ongoing evaluation and refinement throughout implementation. This formative framework facilitated a thorough investigation of how the pilot and initial contact with digital solutions were received by participants [30]. Given health information technology’s increasing role in enabling instant access to critical health services, TPOM’s interconnected flexibility was considered most appropriate [29]. Figure 1 illustrates the TPOM framework, which guided both data collection and analysis, as explained in the following sections.

Within the TPOM, a qualitative approach using reflexive thematic analysis was applied to explore user experiences in depth. Data were gathered via focus groups and semi-structured interviews and analysed via thematic analysis [31, 32]. Arguably, all thematic analysis is inherently reflexive, as even the positivist choice to remain detached is an interpretive decision (Braun & Clarke, 2019). Moreover, for the purposes of this study, a reflexive approach was specifically chosen to allow for the transparent and ethical incorporation of the researcher’s lived experience into the analysis. Further analysis from an organisational and macroenvironmental perspective, is presented in a separate paper from the Here4UScotland remote supervised drug consumption app [25]. Ethical approval was secured from the University of Stirling’s General University Ethical Approval Panel (GUEP; 7800).

## Participants

### Interview, focus group and data collection

Participants were grouped into three categories. Service users (callers) were adults (≥ 18 years) who currently used or had used illicit drugs in the past 12 months and had used or been offered the app while living in Aberdeen. Supporters were harm-reduction–trained community members aged ≥ 18 trained to support app users. Stakeholders included frontline staff, managers, responders, and volunteers involved in harm-reduction or community outreach, alongside local policymakers, Scottish Ambulance Service harm-reduction leads, and police representatives.

Callers and Supports were recruited through the local community partner which was ADA in Aberdeen. Recruitment occurred over period during the pilot, with interviews and focus groups conducted between Month Jun-Nov 2023. The recruitment method was typically convenience and purposive sampling. Stakeholders were invited by CM via email.

All interviews were arranged by email, phone, or in person and conducted by the researcher (GS) either in person, via Microsoft Teams, or by phone, whose lived experience of substance use and associated challenges and stigma, encouraged participant trust and engagement [33]. Callers received a £10 voucher and a debrief sheet outlining study details and support resources.

Two independent online focus groups were held with supporters (*n* = 3) by GS and HD (lasting 60 min). An in-person stakeholder focus group (*n* = 5) was conducted by CM, HD, and GS (1.5 h) in Aberdeen. All were audio-recorded with permission. No demographics were collected due to sensitivities around privacy and unauthorised surveillance. Some participants were from acutely marginalised, stigmatised and vulnerable groups and therefore a decision was made not to ask for or include any personal or social demographics. Data collection ceased once saturation was reached. Interviews were fully transcribed by an external transcriber, with identifiable information removed and Scottish dialect retained. NVivo 12 assisted large-scale data management (GS and HD).

### Data analysis

This study analysed interview data from service users (Supplementary File 1) using the TPOM framework to guide attention toward relevant domains. Semi-structured interviews explored participants’ experiences of the service, including relationships, usability, infrastructure, support needs, and perceived barriers and enablers [31]. We applied reflexive thematic analysis (Braun & Clarke, 2019) to organise the findings within TPOM’s four dimensions and to develop subthemes within each. GS and HD coded all transcripts independently before meeting to compare and refine codes. Reflexive, line-by-line coding was used to capture underlying meanings in participants’ accounts (Braun et al., 2023), and themes were developed through an iterative process of reviewing codes and identifying recurring patterns.

## Results

Findings are presented using the Technology, People and Organisation domains of the TPOM framework, reflecting participants’ accounts of how the Here4UScotland service and smartphones functioned in practice, how they shaped relationships and daily routines, and how organisational structures and responsibilities supported or constrained the service (Table 1).

**Table 1:** Number of recordings, and participants involved

Participants	Main TPOM domains	Method	Participants	Interview or FG group	Method	Sex
Callers	Technological social/human factors	Interviews	8	10 (2 people interviewed twice)	Online & in person	Women = 4 Men = 4
Supporters	Technological, social/human and organizational factors	Focus group (1)	3	3	Online	Women = 2 Men = 1
		Interviews	6	7 (one person interviewed twice)	Online & in person	Women = 4 Men = 2
Community stakeholders	Organizational and wider macro-environment	Focus group (2)	5	5	In person	Women = 3 Men = 2
		Interviews	4	4	Online	Women = 3 Men = 1
Total number			26	29		

## Technology

The Technology domain reflects how participants interacted with the app and devices; within this section, subtheme titles have been adjusted to more clearly convey the central insight of each pattern identified through the analysis. Within the technology component of the TPOM framework, three salient sub-themes emerged: (1) Potential impact of video calling, (2) Location and privacy information, and (3) Usability and connection.

Potential impact of video calling.

Video calling was discussed and suggested as a medium by which people could engage virtually for supervision while using drugs and was viewed positively. It could reduce caller isolation, anxiety and provide reassurance when required:

*I think it would [be beneficial]. … I think it would affect people that have got no families or whatever or maybe couples as well you know maybe you’ve got two people that are using.* (Caller 5).

Women having the opportunity to engage with another female via video calls was viewed positively, contrasting with male involvement. Additionally, face-to-face harm reduction advice could prevent critical outcomes:

*Especially if it’s somebody like you’re seeing face-to-face*,* you know*,* maybe if it’s like a woman or whatever - but you know if you’ve got a female giving you a face-to-face on the internet*,* it’s going to make you feel a bit more comfortable and a bit happier.* (Caller 5).

Video-related digital devices offered security and access to intimate visual cues, potentially facilitating bespoke health information often otherwise unreachable.

### Broad ambivalence on privacy

This reflects patterns also observed in previous evaluations of digital harm reduction tools, which we discuss in greater detail below. Contrary to expectations for vulnerable communities, participants were relatively unconcerned about trusted stakeholders accessing their location:

*When you click on most things on the web now*,* I just click on ‘accept*,*’ so I’ve got no idea what’s coming for me in the next few years with that. But I can’t imagine that you guys are going to use it for anything derogatory at all … *(Caller 2).

This caller’s ambivalence regarding location information highlights their comfort and trust in the service provider. Location tracking was also suggested as a useful measure:

*I’ve had it personally myself where a worker or somebody has been worried about your safety*,* so they’ve sent the police to your house. See if you had a location tracker on you*,* ken*,* that would be an amazing thing*,* I think.* (Caller 6).

However, not all were comfortable, emphasising the importance of user privacy and limited tracking access:

*I could only speculate as to why you know maybe some people wouldn’t want some people coming round to their flat*,* maybe they’re ashamed because of the state their flat is in*,* maybe they’ve got mental health problems where they just can’t speak to people. It could be a number of reasons*,* couldn’t it?* (Caller 5).

Mental health and self-esteem concerns emerged as reasons for apprehension about location availability.

### Improved access and connections

Callers generally found the phones and app straightforward to use. Positive views were expressed regarding access to technology and supporter connection:

*I never actually even thought about*,* oh*,* I’d better actually get that onto loudspeaker or whatever*,* you know. But again*,* that was fairly easy to - you know fairly easy to do. And I tend to just - I’ll make up [prepare] what I’m going to be using*,* and then I’ll phone… *(Caller 2).

The process was understood as easy and appeared to become habitual. Staff assistance further simplified connection and engagement:

*(SM) was with me when I got the phone*,* and I installed it straight away. And within five minutes of me having the phone*,* turning it on*,* connecting to my Wi-Fi*,* the app was installed*. (Caller 1).

This highlights the positive impact of staff-caller relationships, with callers feeling comfortable receiving support. One participant noted this improved connection would be vital if they resumed regular drug use:

*Say I want to start using drugs again and you know I’m needing something like that. Because when I was using drugs*,* I would always use them alone. It would be rare that I would use it with any other people.* (Caller 5).

This emphasised callers’ confidence, even if they had previously experienced isolation due to drug use.

### People

Within the people paradigm, three salient sub-themes emerged regarding the tangible difference digital access made to users: (1) Positive relationships, (2) Finding identity from digital structure, and (3) Safety and security through digital harm reduction.

### Positive relationships through positive digital connections

The evolution of digital services and device access fostered improved relationships, connections, and availability, reducing reliance on analogue face-to-face methods:

*So*,* I think because they’d maybe had conversations with other supporters previously*,* you know*,* they were obviously used to how it worked and how much they were using and the fact that they hadn’t*,* you know*,* had missed their script [prescribed opioid agonist treatment]* (Supporter 3).

This supporter felt digital app interactions provided regular communication and digital cues connected to wellbeing and structure, highlighting a recognised risk from missed prescribed medication. The app provided a safety net. The ability to communicate digitally and instantly with a friendly, non-judgemental voice was also highlighted:

*So*,* I think if you’re getting that non-judgemental attitude on the phone- I think it’s like if the police come in and see drug users*,* they often think I’ll arrest you*,* so then if it’s a friendly person on the phone who understands what’s going on*,* I think that’s a big difference. *(Supporter 4).

While primarily piloted as a virtual drug consumption app, this comment suggests appetite for a broader information and support service to assist users.

### Finding identity from digital structure

Daily digital structure with services offered psychological scaffolding, potentially aiding identity loss and reshaping hopes/ambitions:

*I’m kind of lost in myself*,* and I’m just trying to find more structure and that to my day*,* so it kind of adds to that as well. You know that*,* as I say*,* when I’m using within those times*,* to make use of the app*,* I think it’s a great idea.* (Caller 2)

The caller highlighted feeling psychologically adrift, using the app to improve self-esteem and structure. Digital structure was deemed particularly useful for more systematic, experienced users:

*They’re probably a little bit more on top of their game [(Older users)] than a lot of the younger ones that are just living in the moment*,* blasting snowballs*,* getting arrested and all that…? So*,* I find those ones would be*,* in my opinion*,* more structured in their use*,* more have a bit of a daily routine.* (Supporter 5).

Immediate app access and supporter interaction could provide routine and structure, improving psychological wellbeing.

### Safety and security through digital harm reduction

Digital devices and services offer a neutral medium for individuals with transient, uncertain lives to comfortably access otherwise difficult services:

*I think it’s because clients can almost access you whenever they want*,* and within reason*,* we can pick that up in our working hours. *(Supporter 6).

The reassurance of a digital mediator in reducing anxiety during physical communication was a significant benefit, encouraging engagement. The importance of access for those using alone was also emphasised:

*I spend a lot more time on my own now than I did when I was a wee bit younger*,* just the people – because a lot of the people that are using or whatever*,* you know I don’t particularly want to be you know in touch with or speaking to. So sometimes even just*,* yeah*,* that voice on the end of the line or whatever. (Caller 2).*

Immediate access to harm reduction support, if required, could help callers avoid negative social circles.

### Organisational

The organisational factor within the framework explores interactions between various organisations and new digital services. Three main themes emerged: (1) Cultivating trust, relationships and promoting harm reduction, (2) The gravity of supporter’s responsibility, and (3) Training and service evolution.

### Cultivating trust, relationships and promoting harm reduction

Service providers are responsible for cultivating personal relationships with callers, centred on reciprocal trust and transparent access to harm reduction information via the app. A supporter’s interaction exemplified this:

*So*,* I just kind of checked in with them that they were maybe not going to use as much as they usually would because their tolerance was down. And they were kind of familiar with that already. They were kind of aware of the risks to themselves around that*,* so they said that they weren’t going to use as much. *(Supporter 3).

This reciprocal trust regarding disclosure and harm reduction protocols, by confirming drug dose and acknowledging potential tolerance issues, helps cultivate a risk-averse environment. This success is dependent on relationships, connection and credibility. This was highlighted by repeated calls from the same users:

*I think there’s been a bit of harm reduction*,* definitely a bit of harm reduction. There’s been people who have called more than once so there’s that kind of relationship building with the agency*,* with the service. *(Supporters focus group).

Providing harm reduction information via this audio platform, while maintaining friendly and trusting relationships, is critical for success and fosters a community feel.

### The gravity of responsibility

Supporters providing client support solely via audio operate without crucial visual and social cues. This creates a great deal of post-call anxiety as illustrated by a supporter:

*You go off that phone thinking*,* oh*,* that was okay*,* that went really well. I feel quite content. Imagine you came back in the next day*,* and someone says*,* “Oh*,* Joe Bloggs actually had another overdose*,*” or he died. “What time at?” “1:25.” I’d be like*,* what time was my call? Oh. You know what I mean. 12:45. But they’ve used again in that time. So*,* I think we’re putting a massive lot of responsibility onto supporters here. I think it really needs something (Training) a bit more robust*,* to be honest. *(Supporter 2).

The supporter advocated for more robust and proactive training due toemotional conflict related to the overwhelming responsibility [34].

A participant also suggested broader and wider access to more acute support services:

*I’m not sure at the minute. I think I’d try and use it when I’m alone*,* maybe in a depressed state*,* I don ‘t know. Lifesaving benefits.(It has). Overdose. Suicidal. I’m on the spot here*,* I don’t know. Yeah. Yeah*,* yeah*,* it might be like an intervention type of thing as well.* (Caller 3).

The suggestion of intervention or support also requires more extensive training for supporters.

### Experienced supporters trained in video calling

The lack of training for audio-only support during immediate drug use, without visual cues, was raised by several participants:

*I think probably there would need to be a little bit more training if we were looking for further volunteers*,* maybe out with that kind of specialist role. Especially around*,* you know*,* the signs and symptoms of an overdose because I think when you can see somebody overdosing it’s quite evident but if it’s on the other side of a phone there maybe needs to be a little bit more training around kind of listening out for the signs*. (Supporter 3).

Employing trained supporters with lived experience and familiarity with colloquial language was viewed positively as a solution. This could be further enhanced by adding a video calling feature:

*I think video calling*,* one*,* because people can say whatever they can say but body language is everything and in recovery we ken [know] when - another addict would pretty much know face-to-face when somebody if they’re lying or not*,* ken. So*,* aye*,* I think this would be a good thing*. (Caller 6).

The participant suggested that individuals experienced with drug use can identify signs unfamiliar to others. Video calling enables a more extensive situational assessment.

### Macro-environmental

Although less prominent, macro-environmental factors such as policing and wider policy still shaped participants’ willingness to use the service. These structural influences affected feelings of safety and trust, so a brief summary is retained to reflect their relevance within the TPOM framework.

### Police and emergency services intervention

UK emergency services must advise police when called to potential overdoses, unlike more successful services in Canada and the USA [19, 20]. Scottish participants were understandably concerned about police involvement:

*I’ve certainly heard stories about somebody that had an overdose and then the police administered a log*,* so the ambulance was on the way and then the police officer searched that person and to detained them [presumably due to possession of drugs].* (Stakeholders FG).

Macro concerns were largely limited to police and ambulance, primarily focused on how services might be utilised by authorities for non-public health matters.

## Discussion

This research investigated the impact of new digital devices and services on service users and providers during the Here4UScotland pilot. The TPOM framework informed our interpretation by highlighting how technology, relationships, and organisational factors jointly shaped participants’ experiences. Both parties perceived this technology as beneficial for building connections, community, relationships, and improving outcomes. However, concerns arose regarding the lack of visual cues during potential drug injection or overdose, the need for appropriate training when engaging with people who use drugs (PWUD), and potential inappropriate use of powers by police. Participants showed broad ambivalence about privacy, aligning with previous research indicating marginalised groups’ willingness to engage digitally despite surveillance concerns for learning, knowledge, and relationships [34–36]. Participants warmly discussed the enhanced connections, communication, and engagement opportunities through digital mediums. This echoes previous Scottish and Canadian studies that found digital communication highly beneficial for similar cohorts facing similar issues [37–39]. Such communication is vital for solitary PWUDs, as isolation and vulnerability are key drivers of drug-related fatalities [24, 26–28]. However, the significant responsibility associated with this critical contact can heavily burden volunteer supporters, exacerbated by the lack of visual cues, necessitating specialised medical training for callers. Internationally, Brave Coop in Canada offers extensive training and psychological support for supporters, preparing them for all eventualities [21, 28].

An appetite for digital solutions, particularly video calling, was evident due to reasons like access to visual cues, reassurance, increased connection, bespoke health information, and privacy. Adding this to the current audio platform could significantly enhance its appeal. Additionally, the rapid development of Artificial Intelligence (AI) technology is producing a paradigm shift in digital harm reduction, moving beyond generic interactions. While Here4UScotland represents a vital first step, AI could revolutionise services by offering highly tailored and bespoke support that could analyse individual patterns, preferences, and risk factors, in real-time, to provide responsive, personalised interventions 24/7/365 [39]. This capability extends beyond simply connecting users; it could proactively offer personalised coping strategies, predict risks based on real-time data from integrated wearables, or even identify optimal support networks. This individualised, continuously adaptive model offers a profound evolution from current human-mediated services. However, this advancement is not without significant challenges: ethical, security, privacy, financial, and informed consent concerns are critical considerations for this AI evolution [40, 41].

Privacy and confidentiality were important but less critical than anticipated, consistent with previous research [25, 37]. This might stem from the small cohort size, pre-existing relationships with the host service, and various environmental, cultural, and social factors [42]. Individual attitudes towards privacy were subjective and commonly differ across digital platforms. The potential role of a lived experience researcher in lending credibility to this academic study involving a marginalised group is also worth highlighting [33, 43].

The evolution of service provision from face-to-face to digital communication was welcomed by callers and supporters, who gained immediate access to people and information previously unavailable. Supporters’ experience and empathy towards substance use were considered crucial for overall credibility by both callers and supporters. Immediate access to phone-based digital support positively impacted well-being, offering reassurance, information, and support to individuals accustomed to marginalisation.

### Strengths and limitations

Digital harm reduction is a new concept for most participants, and while uptake of the app was not as popular as hoped, it successfully laid a foundation for future digital harm reduction in Scotland. Here4UScotland was the UK’s first incarnation of remote supervised consumption, serving as a bellwether for all future research in this area. However, the concept requires further evolution to bridge the cultural distance between the UK and more established digital environments in Canada and the USA. Limitations include the study’s location within a smaller Scottish city and a modest cohort size, recruited through a mixture of purposeful and convenience sampling. Additionally, pre-existing relationships between participants and some supporters may have introduced response bias. The pilot was also hindered by a short timeline and a lack of comprehensive project visibility within the area.

## Conclusion

Digital harm reduction technology offers supporters and callers immediate engagement with multiple services, previously unattainable. Unlike historical methods requiring navigation of social anxieties around face-to-face contact, digital access mitigates these obstacles and appears welcome. Despite challenges and subtle interaction within a small community, there was a clear appetite for both digital access and improved harm reduction services.

## Data Availability

No datasets were generated or analysed during the current study.

## References

[1] United Nations Office on Drugs and Crime. World Drug Report 2023 [Internet]. 2023 [cited 2025 May 25]. Available from: https://www.unodc.org/unodc/en/data-and-analysis/world-drug-report-2023.html

[2] European Union Drugs Agency. European Drug Report 2024: trends and developments [Internet]. Lisbon: European Union Drugs Agency; 2024 [cited 2025 Jun 29]. Available from: https://www.euda.europa.eu/publications/european-drug-report/2024_en

[3] Ahmad FB, Rossen LM, Sutton P. Provisional drug overdose death counts. Natl Centre Health Stat. 2021;12.

[4] Fischer B, Pang M, Jones W. The opioid mortality epidemic in North america: do we understand the supply side dynamics of this unprecedented crisis? Subst Abuse Treat Prev Policy. 2020;15(1). 10.1186/s13011-020-0256-8. 10.1186/s13011-020-0256-8 PMC7027114 32066470

[5] Centres for Disease Control and Prevention. U.S. overdose deaths decrease in 2023, first time since 2018 [Internet]. 2024 May 15 [cited 2024 Aug 22]. Available from: https://www.cdc.gov/nchs/pressroom/nchs_press_releases/2024/20240515.htm

[6] Government of Canada. Opioid- and stimulant-related harms in Canada [Internet]. Health Infobase. 2024 Sep 13 [cited 2024 Sep 13]. Available from: https://health-infobase.canada.ca/substance-related-harms/opioids-stimulants/

[7] Kolla G, Touesnard N, Gomes T. Addressing the overdose crisis in North America with bold action. Addiction. 2022;117(5):1194–6. 10.1111/add.15844. 35373484 10.1111/add.15844

[8] BC Coroners Service. More than 2500 lives lost to toxic drugs in 2023 [Internet]. BC Gov News. 2024 Jan 31 [cited 2025 Jul 4]. Available from: https://news.gov.bc.ca/releases/2024PSSG0001-000069

[9] Drug-related deaths in Scotland. 2024 (no date) National Records of Scotland (NRS). Available at: https://www.nrscotland.gov.uk/publications/drug-related-deaths-in-scotland-2024/ (Accessed: 30 October 2025).

[10] Drug misuse deaths increase (no date) National Records of Scotland (NRS). Available at: https://www.nrscotland.gov.uk/latest-news/drug-misuse-deaths-increase/#:~:text=The%20rate%20of%20drug%20poisoning,available%20for%20across%20the%20UK (Accessed: 30 October 2025).

[11] Transform Drug Policy Foundation. Never Use Alone: overdose prevention phone lines [Internet]. 2020 Aug 26 [cited 2025 Jul 4]. Available from: https://transformdrugs.org/blog/never-use-alone-overdose-prevention-phone-lines

[12] NHS Inform. Drugs: what you need to know [Internet]. [place unknown]: NHS Inform; [date unknown] [cited 2024 Nov 1]. Available from: https://www.nhsinform.scot/healthy-living/drugs-and-drug-use/what-you-need-to-know-about-taking-drugs/

[13] Glasgow City Health and Social Care Partnership. Safer Drug Consumption Facility [Internet]. Glasgowcity.hscp.scot. 2024 [cited 2025 May 25]. Available from: https://glasgowcity.hscp.scot/sdcf

[14] Foreman-Mackey A, Bayoumi AM, Miskovic M, Kolla G, Strike C. It’s our safe sanctuary: experiences of using an unsanctioned overdose prevention site in Toronto, Ontario. Int J Drug Policy. 2019;73:135–40. 10.1016/j.drugpo.2019.09.019. 31654936 10.1016/j.drugpo.2019.09.019

[15] Pasman E, Brown S, Agius E, Resko SM. Support for safe consumption sites among peer recovery coaches. J Behav Health Serv Res. 2023. 10.1007/s11414-023-09846-3. 37430133 10.1007/s11414-023-09846-3

[16] Sedaghat N, Seo B, Rider N, Rioux W, Ghosh SM. Perspectives of Canadian healthcare and harm reduction workers on mobile overdose response services: a qualitative study. Subst Use Addict J. 2024;45(3):506–14. 10.1177/29767342241237169. 10.1177/29767342241237169 PMC13021089 38525593

[17] Scottish Drug Deaths Taskforce. Scottish Drug Deaths Taskforce: evidence paper – Final Version [Internet]. 2022 Aug [cited 2025 Jul 4]. Available from: https://drugstaskforce.knowthescore.info/wp-content/uploads/sites/2/2022/08/scottish-drug-deaths-taskforce-evidence-paper-final-version.pdf

[18] Seo B, Kolla G, Touesnard N, Miskovic M, Rider N, Sedaghat N, et al. Perspectives of key interest groups regarding supervised consumption sites (SCS) and novel virtual harm reduction services / overdose response hotlines and applications: a qualitative Canadian study. Harm Reduct J. 2024;21(1):55. 10.1186/s12954-024-00941-z. 39068494 10.1186/s12954-024-01053-3 PMC11282589

[19] Safespot Overdose Hotline. About us [Internet]. 2024 May 14 [cited 2025 May 25]. Available from: https://safe-spot.me/about-us/

[20] National Overdose Response Service (NORS). About. NORS.ca [Internet]. 2024 [cited 2025 May 25]. Available from: https://www.nors.ca/about

[21] Brave.coop. Brave [Internet]. 2025 [cited 2024 Aug 22]. Available from: https://www.brave.coop/

[22] Oteo A, Daneshvar H, Baldacchino A, Matheson C. Overdose alert and response technologies: state-of-the-art review. J Med Internet Res. 2023;25:e40389. 36790860 10.2196/40389 PMC9978985

[23] Perri M, Kaminski N, Bonn M, et al. A qualitative study on overdose response in the era of COVID-19 and beyond: how to spot someone so they never have to use alone. Harm Reduct J. 2021;18(85). 10.1186/s12954-021-00530-3. 10.1186/s12954-021-00530-3 PMC8339679 34353323

[24] Loverock A, Marshall T, Viste D, Safi F, Rioux W, Sedaghat N, et al. Electronic harm reduction interventions for drug overdose monitoring and prevention: a scoping review. Drug Alcohol Depend. 2023;250:110878. 10.1016/j.drugalcdep.2023.110878. 37441959 10.1016/j.drugalcdep.2023.110878

[25] Daneshvar H, Strachan G, Matheson C. Evaluation of the Here4U Scotland application. Digital Lifelines Scotland [Internet]. 2023 [cited 2025 May 25]. Available from: https://digitallifelines.scot/media/1305/evaluation-of-here4u-v6-final.pdf

[26] Strachan G, Daneshvar H, Carver H, Matheson C. Using digital technology to reduce drug-related harms: a targeted service users’ perspective of the digital lifelines Scotland programme. Harm Reduct J. 2024;21(128). 10.1186/s12954-024-01012-y. 10.1186/s12954-024-01012-y PMC11218389 38951880

[27] Daneshvar H, Carver H, Strachan G, Greenhalgh J, Matheson C. From digital inclusion to digital transformation in the prevention of drug-related deaths in scotland: qualitative study. J Med Internet Res. 2024;26:e52345. 39316786 10.2196/52345 PMC11462095

[28] Welwean RA, Krieg O, Casey G, Thompson E, Fleetham D, Deering T, et al. Evaluating the impact of brave technology co-op’s novel drug overdose detection and response devices in North america: a retrospective study. J Urban Health. 2023;100(5):1043–7. 37670172 10.1007/s11524-023-00779-y PMC10618129

[29] Cresswell K, Williams R, Sheikh A. Developing and applying a formative evaluation framework for health information technology implementations: qualitative investigation. J Med Internet Res. 2020;22(6):e15068. 32519968 10.2196/15068 PMC7315366

[30] Dumbrell J, Daneshvar H, Oteo A, Baldacchino A, Matheson C. The acceptability of overdose alert and response technologies: introducing the TPOM-ODART framework. Harm Reduct J. 2023;20(40). 10.1186/s12954-023-00763-4 10.1186/s12954-023-00763-4 PMC10040083 36967388

[31] Braun V, Clarke V. Using thematic analysis in psychology. Qual Res Psychol. 2006;3(2):77–101. 10.1191/1478088706qp063oa.

[32] Molina-Azorín JF. Mixed methods research: an opportunity for methodological advancement. Organ Res Methods. 2016;19(3):329–31.

[33] Francia L, Berg A, Lam T, Morgan K, Nielsen S. The peer workers, they get it–how lived experience expertise strengthens therapeutic alliances and alcohol and other drug treatment-seeking in the hospital setting. Addict Res Theory. 2023;31(2):106–13.

[34] Viste D, Rioux W, Cristall N, Orr T, Taplay P, Morris-Miller L, et al. Association of drug overdoses and user characteristics of canada’s National mobile/virtual overdose response hotline: the National overdose response service (NORS). BMC Public Health. 2023;23(1):1869. 10.1186/s12889-023-16751-z. 37752527 10.1186/s12889-023-16751-z PMC10523711

[35] Gangadharan SP. The downside of digital inclusion: expectations and experiences of privacy and surveillance among marginal internet users. First Monday. 2015;20(2). 10.1177/1461444815614053

[36] Matheson C, Daneshvar H, Carver H, Strachan G, Greenhalgh J. Digital health interventions to prevent drug-related deaths: a rapid review of the evidence. Harm Reduct J. 2023;20(1):110.37587466

[37] Matheson C, Daneshvar H, Carver H, Strachan G, Greenhalgh J, Schofield J. Evaluation of the Digital Lifelines Scotland Programme. Edinburgh: Scottish Government; 2023 Mar [cited 2025 Jul 4]. Available from: https://digitallifelines.scot/media/1217/digital-lifelines-scotland-evaluation-march-2023.pdf

[38] Marshall T, Viste D, Jones S, Kim J, Lee A, Jafri F, et al. Beliefs, attitudes and experiences of virtual overdose monitoring services from the perspectives of people who use substances in canada: a qualitative study. Harm Reduct J. 2023;20(1):80. 37355610 10.1186/s12954-023-00807-9 PMC10290798

[39] Claborn KR, Creech SK, Conway FN, Clinton NM, Brinkley KT, Lippard E, Ramos T, Samora J, Miri A, Benzer J. (2022). Development of a digital platform to improve community response to overdose and prevention among harm reduction organizations. Harm Reduction Journal, 19(1), p.62. 10.1186/s12954-022-00636-2 10.1186/s12954-022-00636-2 PMC9164184 35658871

[40] Carpenter D, Ezell C. An FDA for AI? Pitfalls and plausibility of approval regulation for frontier artificial intelligence. Proc AAAI/ACM Conf AI Ethics Soc. 2024;7(1):239–54.

[41] Machado H, Silva S, Neiva L. Publics’ views on ethical challenges of artificial intelligence: a scoping review. AI Ethics. 2025;5(1):139–67.

[42] Gerber N, Gerber P, Volkamer M. Explaining the privacy paradox: a systematic review of literature investigating privacy attitude and behaviour. Comput Secur. 2018;77:226–61. 10.1016/j.cose.2018.04.002.

[43] Cioffi CC, Hibbard PF, Hagaman A, Tillson M, Vest N. Perspectives of researchers with lived experience in implementation science research: opportunities to close the research-to-practice gap in substance use systems of care. Implement Res Pract. 2023;4:26334895231180635. 37790184 10.1177/26334895231180635 PMC10326466

